# Pregnant Women Who Smoke May Be at Greater Risk of Adverse Effects from Bushfires

**DOI:** 10.3390/ijerph18126223

**Published:** 2021-06-08

**Authors:** Ratika Kumar, Parivash Eftekhari, Gillian Sandra Gould

**Affiliations:** School of Medicine and Public Health, College of Health, Medicine and Wellbeing, The University of Newcastle, Callaghan, NSW 2308, Australia; parivash.eftekhari@newcastle.edu.au (P.E.); gillian.gould@newcastle.edu.au (G.S.G.)

**Keywords:** bushfires, wildfires, pregnancy, smoking

## Abstract

Bushfires substantially increase the environmental health risks for people living in affected areas, especially the disadvantaged (e.g., those experiencing health inequities due to their socio-economic status, racial/ethnic backgrounds, geographic location and/or sexual orientation) and those with pre-existing health conditions. Pregnant women exposed to bushfire smoke are at a greater risk of adverse pregnancy and foetal outcomes, especially if they smoke tobacco, which may compound the toxic impacts. Bushfires may also exacerbate mental stress, leading to an increase in smoking. There are gaps in the evidence and more research is required on the combined effect of bushfire smoke and tobacco smoke on pregnant populations.

## 1. Introduction

Bushfires (e.g., the bushfire crises in several Australian states during 2019–2020) pose a substantial environmental health risk for the populations living in the affected areas. Bushfires are a type of wildfire that occur in “the bush”, which is a collective term for forest, scrub, woodland, or grassland in Australia and New Zealand. Bushfire smoke, which contains noxious gases and particulate matter (PM2.5 and PM10 particles, nitric oxides, carbon monoxide (CO) and other toxic gases), are a substantial health risk and may trigger cardiovascular, respiratory, and inflammatory health events [1]. During December 2019, New South Wales and Australian Capital Territory recorded the highest PM2.5 concentrations in the past two decades (2000–2019) at 26.0 μg/m^3^ and 71.6 μg/m^3^, respectively [2]. Data obtained from Sydney spanning 13.5 years suggest that compared to non-event days, bushfire smoke event days are associated with a 5% increase in non-accidental mortality (OR 1.05, 95% CI 1.00–1.10) [3]. The Australian bushfires of 2019–2020 caused 417 excess deaths due to smoke exposure, 1305 asthma emergencies, and 3151 hospital admissions for cardiovascular and respiratory conditions [4].

Pregnant women may be especially susceptible to bushfire smoke due to physiological changes during pregnancy and likelihood of adverse effects on the foetus, especially if they smoke or live in a smoking household. Since tobacco smoke and bushfire smoke have similar constituents, we explored the risks related to concomitant exposures. The article aims to highlight the adverse health effects experienced by pregnant women living in bushfire-affected areas, especially the effects on their foetus and smoking behaviours, and comment on the research needed to minimise the previously mentioned adverse effects.

## 2. Bushfires and Pregnancy

Very few published studies have explored the direct effect of bushfire or wildfire smoke inhalation on pregnancy and birth outcomes. Due to the highly toxic nature of the exposures, evidence has relied on natural experiments and cohort studies, with or without a control or comparator group [5,6]. Pregnant women exposed to wildfires are at a greater risk of adverse obstetric and birth outcomes, such as pre-term birth, low birth weight, gestational diabetes, and hypertension during pregnancy [6]. Evidence is strongest for birthweight aberrations. A retrospective cohort study, analysing 6147 singleton live births and different levels of bushfire smoke exposure, found that pregnant women who were exposed to high levels (quartile 4) of PM2.5 from burning organic matter in their second or third trimester experienced 1.5 times greater odds of giving birth to children with low birth weight (95% CI, 1.05–2.16), compared to low levels of PM2.5 (quartile 1) [7]. Although clinically insignificant, another study found that babies exposed to bushfire smoke in-utero in any trimester (n = 138,444) weighed 6.1 g less at birth (5% CI: −8.7, −3.5) than unexposed infants (n = 747,590) [5]. Informed by laboratory studies, Holstius et al. proposed key mechanisms for these detrimental effects [5]. These included hypoxia and/or oxidative stress from exposure to constituents of bushfire smoke, including CO and particulate matter, changes in maternal–placental exchanges and endocrine disruption, leading to changes in maternal host–defence mechanisms [5]. Although not from bushfire studies, evidence from systematic reviews suggested that pregnant women’s exposure to air pollutants (similar to bushfire smoke) could result in pre-term and small for gestational age births [8,9]. A recent prospective cohort study showed that each 10 μg/m^3^ increase in mean daily PM2.5 exposure in pregnancy can increase the likelihood of children developing wheeze by 56% (RR, 1.56; 95% CI, 1.18–2.07) and cold or flu by 35% (RR, 1.35; 95% CI, 1.14–1.60) in early childhood [10]. While most studies have reported low birth weight as a result of exposure to bushfires, an Australian study, which analysed data from ACT during the 2003 Canberra wildfires, reported macrosomia (greater birth weight) among male children born to women exposed to bushfires during pregnancy [11]. This increased birth weight was attributed to maternal glucose dysregulation due to crisis-induced increased maternal stress, increased intake of high-calorie nutrient-poor diet, and a lack of adequate exercise (due to official advice to stay indoors or relocate) [11].

## 3. Bushfires, Pregnancy and Smoking

Adverse birth outcomes (e.g., low birth weight and preterm births) have been long associated with active and passive tobacco smoking among pregnant women. These adverse effects in pregnant women are very similar to the exposure to air pollutants from bushfire smoke [8,9,12]. Smoking and bushfires generate similar hazardous air pollutants and may have cumulative effects when combined. An experimental study predicted that at the end of an eight-hour firefighting shift, carboxyhaemoglobin saturation from CO exposure would be 5% in smokers and 3% in non-smokers [13]. It can thus be assumed that the health of populations with a higher prevalence of tobacco smoking and exposure to passive smoking, such as Indigenous People, rural and remote communities, and mental health clients may be disproportionately affected by exposure to bushfires. For the same reason, pregnant women who smoke or are exposed to second-hand smoke may have an overall increase in dose of exposure to such environmental pollutants during bushfires.

### 3.1. Bushfire Trauma and Smoking

Bushfires are also associated with adverse mental health effects. A longitudinal survey of 2063 young adults found an association between the experience of traumatic bushfire-related incidents (and hence greater psychological stress) and increased levels of tobacco smoking (OR 1.12, 95% CI 1.03–1.21), as well as uptake of smoking (OR 1.11 95% CI 1.01–1.23) by non-smokers [14]. As far as we could determine, no studies have explored the relationship between bushfires and smoking behaviours in a pregnant population. However, stress, anxiety, and depression have been linked to difficulties in quitting smoking during pregnancy and relapse to smoking after childbirth [15]. The level of stress increases when the number of stressors increase [16]. The 2019–2020 bushfires in Australia overlapped with the Covid-19 pandemic in early 2020 and might have influenced smoking behaviours of pregnant women due to increased stress [17]. Therefore, a significant evidence gap exists, and bushfire events may warrant exploration about their impact on smoking behaviours during pregnancy.

### 3.2. Effects on Current Smoking Cessation Research

Bushfire smoke may be problematic for a wide range of health research, especially smoking related trials that are evaluating smoking cessation or relapse prevention interventions. Exhaled CO measurement is a frequently used method for biochemically verifying smoking abstinence in smoking cessation clinical trials. However, the large bushfires in a vicinity may affect the validity and reliability of CO monitoring due to those enrolled in the study inhaling CO from the fires and as a result exhaling more CO than what can be attributed to their smoking alone. In such cases, if adequate guidance is not provided to on-ground research personnel and the participants, results of the study may overestimate smoking and incorrectly label non-smokers or ex-smokers as smokers.

## 4. Opportunities for Further Research

High quality evidence on the effects of bushfires on human pregnancy outcomes is lacking. We also do not know the best ways to mitigate these adverse effects. The 2019–2020 Australian bushfires provided an opportunity (albeit tragic) to understand more about the impact of smoke pollution on the natural course of pregnancy for exposed women. Observational studies (cohorts or case-controls) would help investigate the impacts of bushfires on pregnancy outcomes and smoking behaviours. Additionally, qualitative research is required to understand the relationships between exposure to bushfires and their effect on women’s mental health and smoking behaviours.

## 5. Recommendations to Reduce the Health Effects of Smoke from Bushfires in Pregnancy

Although there is no fail-safe way to eliminate the health effects of bushfire smoke, changing individuals’ behaviours may reduce harm. Various health agencies recommend protective measures for pregnant women; however, the evidence base of many recommendations is uncertain as they are based on expert opinion. However, recommendations related to smoking cessation advice are derived from high quality evidence, generated through systematic reviews and meta-analyses of RCTs [18]. As with any behaviour change, public compliance rates may be low, and better public health messaging or media campaigns may be warranted. Additionally, the financial costs of these recommendations may be prohibitive for disadvantaged populations, some of whom may have lost their homes. During Bushfire season or in the event of a bushfire nearby, pregnant women may benefit from limiting outdoor activities, wearing P2/N95 masks when outdoors and preventing entry of smoke inside the home by keeping doors and windows closed. They can also reduce concomitant exposure to other sources of smoke by avoiding smoking cigarettes and cannabis and asking smokers to smoke outside. They can seek assistance to quit from their health providers or Quitline.

## 6. Conclusions

Wildfires or bushfires are becoming a global problem and Australian bushfires could help inform wildfire research in general. The 2019–2020 bushfire season presented Australia with unique challenges for its high-priority populations, especially those pregnant women who were concurrently exposed to bushfire and tobacco smoke. More research is needed to generate high quality evidence about the effects of bushfire smoke pollution in pregnancy, how these exposures can be mitigated, and build clinical guidelines for health practitioners with pregnant clients. The cohort of 2019–2020 pregnant women should be followed up to better understand the combined impacts of bushfires on the physical and mental health of pregnant women, especially those who are smokers or exposed to second-hand smoke.

## Data Availability

Not applicable.
